# Sex-specific depressive symptoms as markers of pre-Alzheimer dementia: findings from the Three-City cohort study

**DOI:** 10.1038/s41398-019-0620-5

**Published:** 2019-11-11

**Authors:** Joanna Norton, Isabelle Carrière, Karine Pérès, Audrey Gabelle, Claudine Berr, Karen Ritchie, Marie-Laure Ancelin

**Affiliations:** 1 Inserm, U1061 Montpellier, France; 2 Montpellier University, Montpellier, France; 3 Inserm, U1219 Bordeaux, France; 4 Bordeaux University, Bordeaux, France; 5 Memory Research and Resources Center, Department of Neurology, CHU Montpellier, Montpellier, France; 6 Centre for Dementia Prevention, Centre for Clinical Brain Sciences, University of Edinburgh, Edinburgh, UK

**Keywords:** Depression, Scientific community

## Abstract

Late-life depression, as a potential marker of pre-dementia, has seldom been explored by symptom dimension and sex, despite sexual dimorphic differences. This study aimed to examine whether specific depressive dimensions were associated with pre-Alzheimer’s disease dementia (pre-AD), separately for women and men. Data were drawn from 5617 (58% women) community-dwellers aged 65+ recruited in 1999–2000 and followed at 2-year intervals for 12 years. We used Cox proportional hazard models to study associations between time-dependent Centre for Epidemiologic Studies-Depression Scale (CES-D) symptom dimensions (namely somatic, depressed, positive affect, and interpersonal challenge) and pre-AD, defined retrospectively from validated diagnoses established 3.5 (IQR: 3.2–4.0) years onwards. Analyses were performed according to overall depressive symptomatology (DS+: CES-D score ≥ 16) and antidepressant/anxiolytic medication use (AA). Results indicated that in DS+ women only, all four dimensions were significantly associated with pre-AD in the AA- group, in particular somatic item ‘Mind’ and depressed affect items ‘Depressed’ and ‘Blues’. The most depression-specific dimension, depressed affect, was also significantly associated with pre-AD in the DS– AA- women (HR:1.28, 95%CI: 1.12;1.47). In both sexes, in the DS– groups somatic affect was the most robust pre-AD marker, irrespective of treatment (women: HR = 1.22, 95%CI: 1.08;1.38; men: HR = 1.30, 95%CI: 1.14;1.48). Our findings highlight sex-specific associations between depressive symptom dimensions and pre-AD, modulated by depressive symptomatology and treatment. Assessment of specific symptom dimensions taking into account overall symptomatology and treatment could help identify and target high-risk AD-dementia profiles for interventions.

## Introduction

Depressive symptoms are common in elderly people and depression is known to affect up to 50% of persons with Alzheimer’s disease (AD) dementia^1,2^. The temporal course of depression, cognitive impairment and dementia are known to be closely related^1–4^, with an ongoing debate as to the nature and direction of these associations^4^.

Evidence points to an association between late-life rather than early or mid-life depression and dementia^5–7^, especially in the case of AD^8^. Findings suggest that depression acts as a marker of pre-dementia rather than as a risk factor per se, potentially sharing common underlying causes such as vascular disease or inflammatory processes^5–7,9,10^. Several prospective studies suggest that late-life depressive symptoms assessed mostly using the Centre for Epidemiologic Studies-Depression Scale (CES-D) may be associated with an increased dementia risk within a narrow time-frame (between 5 and 10 years) preceding dementia onset^6,9,11–13^.

Because the duration of the prodromal phase is uncertain, the time-window in which to consider persons with dementia as pre-demented needs to be addressed^13^. This is crucial to distinguish depressive symptoms as markers of the prodromal phase of disease from those (i) too distant from early symptom onset or (ii) accompanying the early clinical phases of disease with the possibility of reverse causality^14^. So far, differences in study methods with variability both within^15,16^ and between^6,9^ studies in the time-span between depression assessment and dementia onset, have made it difficult to compare findings. Furthermore, studies do not always distinguish between the different sub-types of dementia. One study suggested differential relationships between late-life versus late-onset depressive symptoms and dementia sub-types. Indeed, chronic late-life depression was linked to a greater risk of vascular dementia and late-onset (with no past) depression to a greater risk of AD dementia^8^. Different underlying mechanisms could be involved: late-onset depression may be more likely to reflect a prodromal phase of AD whereas late-life depression would fit better with both the HPA mechanism and vascular-depression dementia hypotheses. Another cause of concern is that potential sources of bias such as antidepressant treatment are not always considered. Yet residual depressive symptoms due to poor treatment response should be distinguished from depressive symptoms in untreated patients.

Whereas the link between depression and dementia has been widely explored, few studies have investigated individual depressive symptoms or dimensions^16–18^. Studies in older adults suggest differential associations between specific depression dimensions and various health outcomes, such as cardio-vascular disease^19,20^ and cognitive functioning^21,22^. Clinically it has been argued that depression in dementia may have different features from that in non-demented persons^17,23,24^. It would be characterised by diminished motivation rather than affective symptoms, such as sadness and guilt^23^. In a 5-year follow-up study of elderly participants with no past depression, ‘loss of interest’ was found to be the only symptom associated with AD onset^16^. In a more recent study, the cognitive/motivational depression dimension predicted dementia onset, but in non-depressed participants only. This suggests a differential symptom effect according to clinical depression status^18^. However, neither of these studies considered depressive symptoms or other covariates as time-dependent variables nor defined a specific time-window for pre-dementia.

Longitudinal studies investigating the dynamic link between depression and dementia separately in men and women are scarce and have used global measures of depression only^7,10,25^. Yet, there is evidence that in non-demented persons the type of depressive symptoms varies considerably between the sexes^26–31^. Women tend to declare more somatic symptoms, such as loss of appetite and fatigue^28,29^, whereas men are more likely to see their life as a failure^31^. Thus, the type of symptoms associated with pre-dementia onset may also vary between the sexes.

The aim of our study was to examine in men and women separately the associations between depression dimensions and pre-AD dementia (pre-AD) in a 12-year longitudinal study of non-institutionalised never-depressed elderly persons. This was performed according to depression status, taking into account overall symptomatology and antidepressant or anxiolytic treatment in an attempt to distinguish the effect of each depressive dimension from that of depression itself.

## Methods

### Study design and participants

Data were drawn from the Three-City Study, a multi-centric prospective cohort study carried out in the French cities of Bordeaux, Dijon and Montpellier^32^. Community-dwelling persons aged 65 and above were recruited randomly from electoral rolls between 1999 and 2000. Participants were followed up at approximately 2, 4, 7, 10 and 12 years. The study protocol was approved by the ethics committees of the University Hospital of Kremlin-Bicêtre and Sud Mediterranée III (Nîmes). All participants gave written informed consent.

At each time-point, participants were administered standardised questionnaires by trained staff with questions on sociodemographic, lifestyle and health characteristics. Patients were asked to report any chronic pathologies from a given list. All drugs used regularly over the past month were recorded and coded according to the Anatomical Therapeutic Chemical Classification (ATC) of the World Health Organisation. To reduce under-reporting, participants were asked to provide both medical prescriptions and drug packages. Fasting blood samples were taken at baseline for apolipoprotein E (APOE) ɛ4 genotyping^11^.

From the original 9294 participants, 216 cases of all-type dementia at inclusion and 296 cases of non-AD dementia at follow-up were excluded. A further 1577 participants with no follow-up data or missing data for AD status at all follow-ups were removed. As pre-AD at time ‘t’ was defined as AD onset between the ‘t + 1’ and ‘t + 2’ follow-ups, 101 participants with AD onset between inclusion and the first follow-up visit were excluded. The analysis was performed on 5617 participants, 2243 men and 3374 women, with no history of major depression (641 excluded) and no missing values for baseline depressive symptomatology and the main covariates (846 excluded) (see Fig. S1, flow chart). Participants excluded from the analysis were more likely to be male, older, APOE ɛ4 carriers, with lower income and education level, more chronic pathologies, visual or hearing impairment, and dependency. They were more likely to have a CES-D score ≥ 16, and use anxiolytic or antidepressant (AA) medication.

### Variables

#### AD and pre-AD dementia

A preliminary diagnosis of dementia and its type was made at each follow-up by the local clinical Three-City Study investigators according to DSM-IV criteria^33^. It was then validated independently by a national panel of neurologists. Date of AD onset was taken to be the midpoint between the diagnosis follow-up and the prior follow-up. In our study, participants were considered pre-demented at time-point ‘t’, two follow-ups prior to the ‘t + 2’ follow-up at which AD diagnosis was established for the first time. This corresponds to a median (IQR) duration of 3.5 (3.2–4.0) years, 3.6 (3.2–4.1) years for women and 3.5 (3.1–4.0) years for men, between pre-AD and diagnosis. This allowed for an intermediate dementia-free time-point (‘t + 1’), in order to ensure a 2-year dementia-free time-lag between depressive symptom assessment and the beginning of the dementia onset period.

#### Depressive symptomatology

Depressive symptoms were assessed at inclusion and each of the follow-ups using the validated 20-item CES-D scale^34^. For each item, participants rated how frequently they applied to them over the past week. Ratings were based on a 4-point Likert scale ranging from 0 (rarely, none of the time) to 3 (frequently, most of the time). High scores on all negatively-worded items reflected more severe symptomatology. For consistency purposes, the four positive-worded items were reversed so that high scores reflected more severe symptomatology. The CES-D is generally considered to have a 4-factor structure consisting of depressed affect, somatic affect, positive affect and interpersonal challenge^26^, although the exact items for each dimension vary somewhat from one study to another^26,34–36^. Following Carleton’s review of previous CES-D validation studies^26^, we chose to use a modified 4-factor version of the original solution^26,34^. Scores on the somatic (seven items) and depressed affect (five items) dimensions were standardised to the 0-to-12 scale of the positive affect and interpersonal challenge dimensions (four items each) for comparability purposes. We also distinguished participants with low and high depressive symptomatology (DS) using the 16+ cut-off on the global CES-D score^37^. The Mini International Neuropsychiatric Interview was used only to identify participants at baseline with a history of major depression according to DSM-IV criteria^38^.

#### Covariates

Baseline variables: socio-demographic factors included age, sex and education (>than 5 years). Health and lifestyle characteristics recorded at baseline only included smoking (never, past, current) and current alcohol consumption (g/day).

Time-dependent variables: these were measured at baseline and each follow-up and included: living situation (alone or not), body mass index (BMI) (calculated from reported height and weight), visual or hearing impairment^39^, and the following chronic diseases: hypertension (≥140/90 mm Hg or treated), hypercholesterolemia and diabetes (self-reported or treated), and any self-reported ischemic disease (angina, coronary angioplasty, cardiac bypass, myocardial infarction, stroke, arteritis). The median score on the Mini Mental State Examination (MMSE) was used as a global measure of cognitive function^40^. A hierarchical 3-level measure of dependency was constructed^41^, combining the Rosow-Breslau mobility scale^42^, the Lawton-Brody Instrumental Activities of Daily Living scale^43^ and Katz Activities of Daily Living scale^44^. Antidepressant (N06A) and anxiolytic (N05B) medication use in the past month were recorded at each time-point.

### Statistical analysis

The sample was described separately for men and women at baseline, using percentages for categorical variables and means (SD) or medians (IQR) for continuous variables, after testing for normality using the Shapiro-Wilk test.

All four depression dimension scores and binary DS were considered independently at baseline and as time-dependent variables. Statistical tests were performed using Cox models with delayed entry, with age as the basic time-scale and birth as the time origin^45^. This method enables a better adjustment for age than the standard model with time since inclusion as the time-scale. Thus, time-points should be understood as age-points. The assumptions of proportional hazards over time for baseline variables and the log-linearity of CES-D scores and other continuous covariates were verified. Results were expressed as hazard ratios (HR) with 95 % confidence intervals (CI). HRs represent the increased risk per one-point increase in score on an ordinal 0–12 scale. For the positive affect and interpersonal challenge dimensions, this directly represents one additional point on the 0–12 symptom scale; for the depressed and somatic affect dimensions, this represents 1.25 and 1.75 additional points on the five-items (0–15 score) and seven-items (0–21 score) scales, respectively. For the total score, this represents a 5-point increase on the 20-item (0–60 score) scale. For each individual symptom however, the increase corresponds to a one-point increase on the 0–3 scale.

Covariates were classified as either fixed and unchanging across the follow-ups or, when data were available, as time-dependent. Covariates associated with pre-AD in either sex with *p*-values < 0.15 were considered for entry in the multivariate analysis (see Table S1). We further examined the relationship between symptom dimensions and pre-AD stratified by DS and AA use. Finally, we examined individual items categorised as binary variables (score: 0/1+), using Bonferroni correction for multiple testing. Statistical analyses were performed using SAS Enterprise Guide Version 7.15 (SAS Institute, Inc. Cary, North Carolina).

## Results

### Sample description

Median (IQR) age at inclusion was 73.2 (69.4–77.2) for women and 72.5 (69.2–76.8) for men. Of the sample, 24.3% of women and 10.6% of men had high DS (Table 1). Median (IQR) follow-up duration was 10.1 (IQR = 6.9–11.3) and 9.1 (6.3–11.2) years, for women and men respectively. There were 294 AD cases at follow-up for the women and 139 cases for the men.

**Table 1:** Socio-demographic, lifestyle and health characteristics of the sample at baseline, for women and men separately^a^

	Women (*N* = 3374)	Men (*N* = 2243)	
	%	%	*p*^d^
Centre			
Bordeaux	23.8	23.1	
Dijon	57.4	53.7	
Montpellier	18.8	23.3	0.0002
Age (years) (median, IQR)	73.2 (69.4–77.2)	72.5 (69.2–76.9)	0.004
Education (>5 years)	75.3	78.6	0.005
Living alone	48.2	13.9	<0.0001
Smoking			
Never	81.9	31.3	
Former	14.4	60.6	
Current	3.7	8.1	<0.0001
Alcohol consumption (g/day) (median, IQR)	4.5 (0–11.0)	19.2 (2.3–30.5)	<0.0001
BMI (kg/m^2^) (mean, SD)	25.4 (4.3)	26.2 (3.4)	<0.0001
Chronic diseases			
Hypertension	73.9	64.1	<0.0001
Hypercholesterolemia	39.4	34.2	<0.0001
Diabetes	5.6	9.9	<0.0001
Ischemic disease	11.3	20.7	<0.0001
MMSE score (median, IQR)	28 (27–29)	28 (27–29)	0.29
Visual or hearing impairment	18.8	18.2	0.55
Dependency level			
Low (or fully independent)	50.0	69.3	
Moderate (mobility restriction only)	42.3	26.0	
High (IADL and/or ADL limitation)	7.7	4.7	<0.0001
Anxiolytic consumption	17.2	7.6	<0.0001
Antidepressant consumption	7.0	2.4	<0.0001
APOE ɛ4 carrier	19.3	20.2	0.40
CES-D dimensions (median, IQR)			
Somatic affect^b^	1.7 (0.6–2.9)	1.1 (0.6–2.3)	<0.0001
Depressed affect^b^	0.8 (0–3.2)	0 (0–0.8)	<0.0001
Positive affect^c^	3.0 (1–6)	2.0 (0–4)	<0.0001
Interpersonal challenge	0 (0–2)	0 (0–1)	<0.0001
Total CES-D score	9 (4–15)	6 (2–10)	<0.0001
Depressive symptomatology: high (CES-D≥16)	24.3	10.6	<0.0001

^a^less than 1% missing values except for alcohol consumption (6.5%)

^b^standardised to 0–12 scale

^c^reversed so that a high score reflects low positive affect

^d^*χ*^2^ test for categorical variables, Student’s *T*-test for normally distributed continuous variables and Wilcoxon Test for skewed continuous or ordinal variables

### Association between depressive symptom dimensions and pre-AD dementia

For women only, all four dimensions were associated with pre-AD. The strongest association was observed for the somatic affect dimension (Table 2). The somatic and depressed affect dimensions were significantly associated with pre-AD irrespective of DS, whereas the remaining two dimensions were associated in the DS+ group only (Table 3). More specifically, in the DS– group, the association for somatic affect was significant in the treated women only. For somatic affect in the AA- women, we removed covariates one by one from the model and the association was significant when excluding time-dependent dependency (*p* = 0.03) (data not shown). In the DS+ group, the four dimensions were significantly associated with pre-AD in the untreated women only. Further adjusting for continuous time-dependent MMSE score did not modify the findings (data not shown).

**Table 2:** Risk of incident AD pre-dementia associated with time-dependent depression dimensions (separate models for each dimension) and depressive symptomatology

	items	HR	(95%CI)	*p*^c^
Women (294 events/3374)				
Depression dimensions				
Somatic affect^a^	(1, 2, 5, 7, 11, 13, 20)	1.08	(1.02;1.14)	0.007
Depressed affect^a^	(3, 6, 14, 17, 18)	1.05	(1.01;1.10)	0.02
Positive affect^b^	(4, 8, 12, 16)	1.04	(1.00;1.08)	0.04
Interpersonal challenge	(9, 10, 15, 19)	1.08	(1.00;1.16)	0.04
Total score		1.10	(1.03;1.07)	0.003
Depressive symptomatology: high	(Total score ≥ 16)	1.39	(1.08;1.80)	0.01
Men (139 events/2243)				
Depression dimensions				
Somatic affect^a^	(1, 2, 5, 7, 11, 13, 20)	1.08	(0.96;1.19)	0.14
Depressed affect^a^	(3, 6, 14, 17, 18)	0.98	(0.88;1.10)	0.72
Positive affect^b^	(4, 8, 12, 16)	1.02	(0.96;1.09)	0.58
Interpersonal challenge	(9, 10, 15, 19)	1.07	(0.92;1.23)	0.41
Total score^a^		1.06	(0.93;1.20)	0.38
Depressive symptomatology: high	(Total score ≥ 16)	0.92	(0.50;1.68)	0.79

^a^standardised to 0–12 scale

^b^reversed so that a high score reflects a low positive affect

^c^Cox proportional hazard models with age as the time-scale, adjusted for study centre, education (>5 years), Apoe4 and the following time-dependent variables: diabetes (no/yes), ischemic disease (no/yes), dependency (3 levels), anxiolytic or antidepressant consumption (no/yes)

**Table 3:** Risk of incident AD pre-dementia associated with time-dependent depression dimensions (separate models for each dimension), stratified by depressive symptomatology and antidepressant or anxiolytic (AA) medication use

	Women (*n* events/*N*)		Men (*n* events/*N*)	
	HR (95% CI)	*p*^e^	HR (95% CI)	*p*^e^
Depressive symptomatology: Low^a^	164/1902		118/1744	
Somatic affect^c^	1.22 (1.08;1.38)	0.002	1.30 (1.14;1.48)	0.0001
Depressed affect^c^	1.15 (1.02;1.29)	0.02	1.01 (0.82;1.25)	0.92
Positive affect^d^	1.05 (0.98;1.13)	0.19	1.02 (0.94;1.12)	0.60
Interpersonal challenge	1.03 (0.86;1.24)	0.74	1.27 (1.02;1.58)	0.03
Total score^c^	1.36 (1.12;1.64)	0.002	1.38 (1.10;1.74)	0.006
AA use: No^b^	**1**12/1432		99/1495	
Somatic affect^c^	1.14 (0.98;1.32)	0.09	1.26 (1.08;1.47)	0.004
Depressed affect^c^	1.28 (1.12;1.47)	0.0004	0.94 (0.73;1.21)	0.62
Positive affect^d^	1.04 (0.95;1.14)	0.36	0.99 (0.90;1.10)	0.99
Interpersonal challenge	1.05 (0.85;1.30)	0.65	1.16 (0.87;1.53)	0.31
AA use: Yes^b^	52/470		19/249	
Somatic affect^c^	1.37 (1.09;1.72)	0.008	1.51 (1.14;1.99)	0.004
Depressed affect^c^	0.97 (0.78;1.21)	0.79	1.59 (1.03;2.45)	0.04
Positive affect^d^	1.08 (0.95;1.22)	0.25	1.12 (0.92;1.37)	0.25
Interpersonal challenge	0.93 (0.66;1.31)	0.68	1.77 (1.22;2.59)	0.003
Depressive symptomatology: High^a^	130/1472		21/499	
Somatic affect^c^	1.11 (1.03;1.19)	0.009	0.97 (0.78;1.20)	0.77
Depressed affect^c^	1.09 (1.03;1.15)	0.002	1.11 (0.95;1.30)	0.20
Positive affect^d^	1.08 (1.02;1.15)	0.005	1.16 (1.01;1.34)	0.03
Interpersonal challenge	1.13 (1.04;1.23)	0.003	1.05 (0.82;1.35)	0.70
Total score^c^	1.18 (1.08;1.29)	0.002	1.15 (0.91;1.45)	0.25
AA use: No^b^	61/714			
Somatic affect^c^	1.17 (1.05;1.31)	0.007	–	
Depressed affect^c^	1.15 (1.07;1.25)	0.0004	–	
Positive affect^d^	1.10 (1.02;1.19)	0.02	–	
Interpersonal challenge	1.26 (1.12;1.41)	0.0001	–	
AA use: Yes^b^	69/758			
Somatic affect^c^	1.08 (0.97;1.20)	0.18	–	
Depressed affect^c^	1.04 (0.96;1.12)	0.34	–	
Positive affect^d^	1.07 (0.99;1.16)	0.08	–	
Interpersonal challenge	1.03 (0.91;1.16)	0.65	–	

^a^for pre-AD dementia subjects: a high versus low depressive symptomatology was defined as a CES-D score ≥ 16 at assessment point or at any earlier follow-up, including study entry; for others: this was defined as a CES-D score ≥ 16 at the 2, 4 or 7-year follow-up

^b^for pre-AD dementia subjects: AA use was defined as use of AA medication at assessment point or at any earlier follow-up, including study entry; for others: this was defined as use at the 2, 4 or 7-year follow-up

^c^standardised to 0–12 scale

^d^reversed so that a high score reflects a low positive affect

^e^Cox proportional hazard model with age as the time-scale, adjusted for study centre, education (>5 years), Apoe4 and the following time-dependent variables: diabetes (no/yes), ischemic disease (no/yes), dependency (3 levels)

For men, the somatic affect and interpersonal challenge dimensions were both significantly associated with pre-AD in the DS– group, irrespective of treatment for somatic affect only; this was also the case for positive affect in the DS+ group. The results were unchanged when further adjusting for MMSE score (data not shown). The effect of AA use could not be examined in the DS+ group due to low number of events (21) and AA users (5).

### Association between individual depressive symptoms and pre-AD dementia

**Fig. 1:**
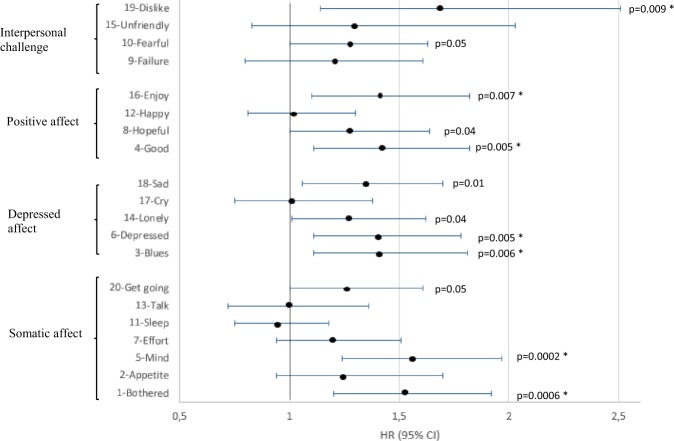
Multi-adjusted associations between individual depression symptoms and pre-AD dementia–women. *significant when applying Bonferroni correction (with *p*-value thresholds: *p* ≤ 0.007 for the somatic dimension and *p* ≤ 0.01 for the depressed affect, positive affect and interpersonal challenge dimensions)

Associations for women between individual items and pre-AD are shown in Fig. 1, with an indication of significance after multiple comparison correction. Associations further differed according to DS and AA use. For instance, somatic affect item ‘Bothered’ remained highly significantly associated with pre-AD in the DS– group only, and more specifically in the AA+ women (see Table S2). Conversely, ‘Mind’, ‘Blues’, ‘Depressed’, ‘Sad’ and ‘Dislike’ were highly significantly associated with pre-AD in the DS+ group, with strongest associations in the untreated women.

**Fig. 2:**
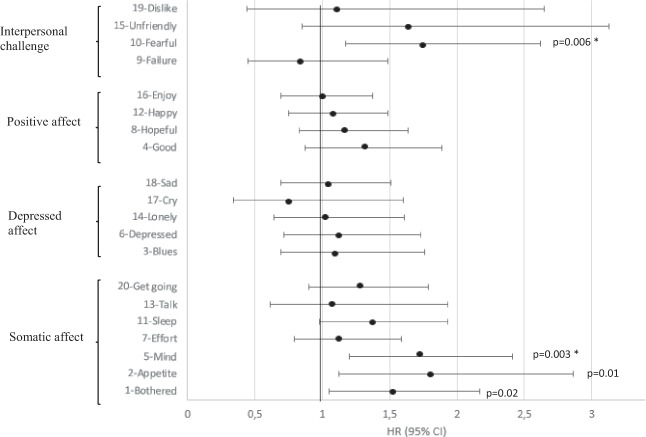
Multi-adjusted associations between individual depression symptoms and pre-AD dementia–men. *significant when applying Bonferroni correction (with *p*-value thresholds: *p* ≤ 0.007 for the somatic dimension and *p* ≤ 0.01 for the depressed affect, positive affect and interpersonal challenge dimensions)

For men, only somatic affect items ‘Mind’ and ‘Fearful’ remained significant after multiple comparison correction (Fig. 2), and they were also highly significant in the DS– group (HR = 2.03 (1.39;2.98), *p* = 0.0003 and HR = 2.37 (1.0;3.74), *p* = 0.0002, respectively).

## Discussion

This is one of the first studies to investigate separately in elderly women and men the association between late-onset depressive symptom dimensions and pre-AD defined retrospectively from expert-panel validated AD diagnoses. Overall, our findings suggest a differential pattern of associations according to sex and depression status, assessed by overall depressive symptomatology and AA use. In high DS women, the four dimensions were significantly associated with pre-AD in the untreated but not in the treated women. This was also the case for depressed affect in the low DS women. In addition, somatic affect stood out as the most robust marker of pre-AD in low symptomatology women and men, whatever their treatment status.

### The pre-dementia phase: methodological considerations and study design

When studying depression as a potential marker of pre-dementia, the definition of the period associated with this phase is crucial as the type and intensity of depressive symptoms may change over time. Based on recent findings from large prospective studies^6,9,46^, we chose to target the 3–4 year time-period before the diagnosis in order to closely capture the pre-AD phase and avoid confounding with the dementia state. To date, most studies show considerable variability in time-to-event even within a restricted time-frame as few have adopted a time-dependent approach to examine the depression-dementia association. Furthermore, to our knowledge, no studies have allowed for an intermediate diagnosis-free time-point between the pre-AD depression assessment and the dementia onset period. The 2-year diagnosis-free time-lag in our study reduced the risk of capturing a concomitant onset of both conditions.

### Depressive symptom dimensions and pre-AD dementia

Few studies have examined depression dimensions with respect to dementia onset^5,16,18^ and the use of different depression scales, with varying numbers and types of symptom dimensions limits the comparability of findings^19^. In a cohort of 3410 elderly participants, Li et al. (2011) found that any score > 0 on the depressed affect dimension of the 11-item CES-D version was associated with a 3-year increased risk of dementia^5^. In a five-year follow-up study of 437 never-depressed elderly, out of the nine DSM-IV-TR depressive symptoms, only ‘loss of interest’ was associated with AD onset^16^. This symptom was not included in the original 20-item CES-D, thus making comparisons spurious. However, neither of these studies took into account time-to-diagnosis or considered depressive symptomatology as a time-dependent variable.

### Sex differences in low versus high DS groups

Sex differences in the association between depression and pre-dementia have seldom been examined. Overall, findings are contradictory as to the existence^6,7,12^ and direction^10,25^ of sex differences in the association between late-life depressive symptoms measured using the CES-D and pre-AD. Conversely, to studies suggesting associations in men but not women^10,25^, we found in a larger dataset with a greater number of incident AD cases an association in women only. These contradictory findings may be explained by differences in the study populations, for example with respect to age, education, and baseline cognitive functioning. Studies also vary in their methodological approach, focusing on late-life rather than late-onset depression and not always allowing for a diagnosis-free time-lag between assessment and dementia onset^10^.

Differential findings in men and women can be expected as they differ with respect to the type and intensity of depressive symptoms, and the 16+ threshold for the CES-D will encompass different symptom profiles in each sex. Furthermore, they differ with respect to symptom reporting, symptom recognition and treatment. Symptoms in men are less likely to be recognised and treated, as shown by the low absolute number of men reporting AA treatment in our study (5 out of 21 DS+ men). Also, men may be less willing to admit to experiencing symptoms, and those scoring high on the CES-D may be reporting symptoms linked to other chronic diseases, rather than depression per se^47^. Differential mortality levels and patterns in men and women over the follow-up will also influence the relationship between depressive symptoms and pre-dementia.

### Sex differences according to depressive dimensions

The overall association found in our study in women but not men hides significant associations in both when examining the symptom dimensions separately in each of the DS categories. This justifies the need to look beyond depressive symptomatology as a single entity binary variable. No studies so far have examined the different symptom dimensions according to sex. We found that in low DS groups, somatic affect was associated with pre-AD in both women and men. Conversely, the most depression-specific dimension, depressed affect, was significant in women only and interpersonal challenge in men only. The greatest differences concerned the high DS group where highly significant associations were found for the four dimensions in women, specifically those untreated.

So far, antidepressant treatment has been mainly analysed as a confounder, usually measured at a fixed time-point. Fuhrer et al. (2003) studied the joint effects of time-dependent depressive symptoms (CES-D score ≥ 23 for women and ≥ 17 for men) and antidepressant medication on AD dementia; with the ‘no depression-no treatment’ group as the reference category, they reported significant associations for all three depression-treatment combinations for men only^10^. However, the low number of participants per group calls for caution in interpreting their findings. Furthermore, physician prescription behaviour and patient treatment may differ between the sexes.

### Depressive symptoms and pre-AD according to depression status

In keeping with studies suggesting a differential pattern of depressive symptoms in subjects with dementia^17^, varying according to depression itself^18^, we examined associations for individual symptoms according to overall DS and AA use. In women with high DS, associations for all four dimensions were restricted to those not taking AA, in particular depressed affect items ‘Depressed’ and ‘Blues’, and somatic affect item ‘Mind’; this was also the case for depressed affect in low DS women. This may suggest a positive effect of AA treatment on cognition, whatever the overall DS. AA medication may potentially help delay dementia onset by altering the mechanisms linking depressive symptoms to dementia: for instance, through shared risk factors such as cerebrovascular disease and inflammation^48^, or a causal effect of depressive symptoms on the dementia process through hippocampal damage^49^. An alternative explanation is that pre-dementia may reduce the likelihood of receiving AA treatment, either because pre-demented subjects do not seek treatment or because symptoms are attributed to other causes, such as cognitive decline.

Somatic affect stood out as the most robust marker of pre-AD in low DS women and men, whatever their treatment status, although time-dependent dependency level seldom considered in other studies as a covariate may have confounded the association for the untreated women. Several individual symptoms were associated with pre-AD, but only ‘Mind’ in both sexes and ‘Bothered’ in women remained significant after multiple comparison correction. This suggests that somatic symptoms potentially attributable to other conditions than depression could be involved. This is in keeping with Lugtenburg et al. (2015) who reported that the cognitive/motivational depression dimension identified using the GMS-AGECAT predicted 3-year incident dementia, but in non-depressed elderly only^18^. They concluded that cognitive and motivational symptoms of depression were likely to reflect cognitive complaints, particularly in the absence of depression.

### CES-D factor structure

Different factor structures have been proposed for the CES-D, varying according to the number of dimensions and dimension-specific items^26^. Contrary to studies which apply Radloff’s original 20-item 4-factor structure^34,36^, we used the modified 4-factor structure (Model [B])^26^, with items 9 (Failure) and 10 (Fearful) classified in the interpersonal challenge rather than depressed affect dimension. Confirmatory factor analysis revealed that this model had one of the best factorial validities^26^. It also had similar factorial validity compared to the original model in our study. We nonetheless reran our analyses using the original 20-item 4-factor structure. Our findings were unchanged except for the two-item interpersonal challenge dimension (score 0/1+), which was no longer significant for DS– men (data not shown).

### Limitations

Several limitations must be taken into account in our study. To start, we relied on the self-report but validated CES-D scale rather than on a psychiatric interview to assess depressive symptomatology. We applied the widely used 16+ cut-off to distinguish subjects with low versus high DS. Despite its good psychometric properties, a CES-D score ≥16 is not equivalent to a clinical diagnosis of major depression^47^. We also repeated our analyses with the French-validated 17+ (for men) and 23+ (for women) thresholds^50^; our findings were largely unchanged. Secondly, in order to capture a more homogenous group of subjects and focus on late-onset depression only, we excluded participants with a history of major depression. Our findings were unchanged, albeit less significant, when performing a sensitivity analysis including all subjects. Thirdly, pre-demented participants may differentially report depressive symptoms, thus modifying the associations with pre-dementia. Fourthly, unmeasured confounding bias is always possible and may have altered our findings. We thus calculated *E*-values for the significant multi-adjusted parameters in Table 2^51^. The strength of the association that an unmeasured confounder would require to ‘explain away’ the observed associations, independently of the many covariates already taken into account, suggests this is unlikely. Extreme caution is nevertheless required for the interpretation of our findings and causality can by no means be assessed. Finally, a difference in attrition with participants who will go on to develop dementia being more likely to be censored than others is a further limitation potentially leading to an underestimation of the associations.

### Strengths

On the other hand, this prospective study was based on a large community sample with six assessments performed over 12 years including a clinical examination at each time-point. The large sample size allowed us to perform our analysis on a homogenous group of pre-AD participants, stratified by sex, despite the lower number of events in men. We were also able to consider overall symptomatology and treatment, and into account a number of time-dependent covariates in our analysis. An additional strength is the validation of probable dementia cases by an expert panel of clinicians, given the low sensitivity of case-records for identifying cases^6,9^.

## Conclusion

Our findings highlight sex-specific associations between depression dimensions, specific symptoms and pre-AD, which vary according to the overall symptom level and treatment. In women, the most depression-specific dimension, depressed affect, was a marker of pre-AD in the absence of treatment only. The least depression-specific dimension, somatic affect, stood out as the most robust marker of pre-AD in both women and men with low symptomatology. Assessment of specific symptoms, as well as overall depressive symptomatology and treatment, could help identify high-risk AD dementia profiles likely to benefit from interventions.

## Supplementary information

Table S1, Table S2

Figure S1
