# Supplementary material for: Sex-specific depressive symptoms as markers of pre-Alzheimer dementia: findings from the Three-City cohort study
Source: Transl Psychiatry. 2019 Nov 11;9:291. doi: 10.1038/s41398-019-0620-5 (PMC6848073; doi:10.1038/s41398-019-0620-5)
Supplement: Supplementary file 1 — Table S1, Table S2 [file 41398_2019_620_MOESM1_ESM.docx]

Supplementary Table 1. Minimally adjusted associations between socio-demographic, lifestyle and health characteristics, and the risk of incident AD pre-dementia.

|  | **Women**  **(294 events/3374)** | |  | **Men**  **(139 events/2243)** | |
| --- | --- | --- | --- | --- | --- |
| **Variables at inclusion:^1^** | **HR (95% CI)** | **P^2^** |  | **HR (95% CI)** | **P^2^** |
| Centre |  |  |  |  |  |
| Bordeaux | 1 |  |  | 1 |  |
| Dijon | 0.8 (0.61;1.02) |  |  | 1.09 (0.75;1.59) |  |
| Montpellier | 0.64 (0.44;0.92) | 0.04 |  | 0.42 (0.23;0.75) | 0.003 |
| Education (> 5 years) | 1.48 (1.16;1.89) | 0.002 |  | 2.14 (1.51;3.04) | <0.0001 |
| Smoking  Never  Former  Current | 1  0.97 (0.68;1.39)  1.59 (0.91;2.78) | 0.26 |  | 1  1.04 (0.72;1.49)  0.84 (0.40;1.79) | 0.85 |
| Alcohol consumption (g/day) | 1.00 (0.99; 1.02) | 0.54 |  | 0.99 (0.98; 1.01) | 0.28 |
| Apo ɛ4 carrier | 2.13 (1.64;2.75) | <0.0001 |  | 1.59 (1.07;2.36) | 0.02 |
| **Time-dependent variables:^1^** |  |  |  |  |  |
| Living alone | 1.14 (0.89;1.46) | 0.28 |  | 0.75 (0.47;1.20) | 0.23 |
| BMI (kg/m^2^) | 0.99 (0.84;1.17) | 0.91 |  | 1.02 (0.79;1.32) | 0.89 |
| Chronic diseases: |  |  |  |  |  |
| Hypertension | 0.97 (0.78;1.22) | 0.79 |  | 0.88 (0.64;1.23) | 0.47 |
| hypercholesterolemia | 1.04 (0.82;1.31) | 0.76 |  | 0.96 (0.68;1.35) | 0.80 |
| Diabetes | 1.66 (1.17;2.35) | 0.004 |  | 1.53 (0.97;2.41) | 0.07 |
| Ischemic disease | 1.28 (0.97;1.69) | 0.08 |  | 1.09 (0.76;1.58) | 0.63 |
| MMSE score | 0.81 (0.77;0.85) | <0.0001 |  | 0.81 (0.75;0.87) | <0.0001 |
| Visual or hearing impairment | 1.01 (0.77;1.31) | 0.95 |  | 1.25 (0.86;1.82) | 0.25 |
| Dependency level  Low  Moderate  High | 1  1.64 (1.17;2.29)  2.52 (1.74;3.67) | 0.004  <0.0001 |  | 1  1.08 (0.74;1.58)  1.18 (0.69;2.02) | 0.83 |
| Anxiolytic or antidepressant use | 1.46 (1.14;1.86) | 0.003 |  | 1.42 (0.91;2.23) | 0.13 |

^1^Less than 0.5% missing values except for alcohol consumption (6% for men; 7% for women).

**^2^**Cox proportional hazard model with age as the time-scale, adjusted for study centre, education (>5 years)

Supplementary Table 2. Risk of incident AD pre-dementia associated with time-dependent individual CES-D items (separate models for each item), stratified by depressive symptomatology and antidepressant or anxiolytic (AA) medication use, in women.

| **Depressive symptomatology^1^** | **Low**  **(164/1902)** |  | **High**  **(130/1472)** |  |  |
| --- | --- | --- | --- | --- | --- |
| **Individual items** | **HR (95% CI)** | **P^2^** | **HR(95% CI)** | **P^2^** |  |
| **Somatic affect** |  |  |  |  |  |
| 1-Bothered | 1.70 (1.22;2.38) | 0.002 | 1.45 (1.03;2.05) | 0.04 |  |
| AA- | 1.49 (0.98;2.27) | 0.07 | - |  |  |
| AA+ | 2.56 (1.44;4.57) | 0.001 | - |  |  |
|  |  |  |  |  |  |
| 2-Appetite | 1.94 (1.28;2.95) | 0.002 | 1.01 (0.68;1.49) | 0.96 |  |
|  |  |  |  |  |  |
| 5-Mind | 1.50 (1.07;2.09) | 0.02 | 1.91 (1.32;2.76) | 0.0006 |  |
| AA- | - |  | 3.37 (1.88;6.04) | <0.0001 |  |
| AA+ | - |  | 1.35 (0.83;2.21) | 0.23 |  |
|  |  |  |  |  |  |
| **Depressed affect** |  |  |  |  |  |
| 3-Blues | 1.28 (0.85;1.93) | 0.23 | 1.99 (1.37;2.88) | 0.0003 |  |
| AA- | - |  | 2.20 (1.30;3.73) | 0.003 |  |
| AA+ | - |  | 1.80 (1.06;3.07) | 0.03 |  |
|  |  |  |  |  |  |
| 6-Depressed | 1.31 (0.89;1.93) | 0.16 | 2.08 (1.40;3.08) | 0.0003 |  |
| AA- | - |  | 3.21 (1.80;5.73) | <0.0001 |  |
| AA+ | - |  | 1.42 (0.83;2.45) | 0.20 |  |
|  |  |  |  |  |  |
| 14-Lonely | 1.35 (0.94;1.92) | 0.10 | 1.48 (1.03;2.13) | 0.04 |  |
| AA- | 1.87 (1.23;2.83) | 0.003 | 2.17 (1.24;3.78) | 0.007 |  |
| AA+ | 0.59 (0.27;1.27) | 0.18 | 1.07 (0.65;1.74) | 0.80 |  |
|  |  |  |  |  |  |
| 18-Sad | 1.34 (0.94;1.92) | 0.10 | 1.81 (1.19;2.73) | 0.0006 |  |
| AA- | - |  | 2.38 (1.26;4.50) | 0.008 |  |
| AA+ | - |  | 1.42 (0.82;2.48) | 0.21 |  |
|  |  |  |  |  |  |
| **Interpersonal challenge** |  |  |  |  |  |
| 9-Failure | 1.23 (0.72;2.11) | 0.44 | 1.36 (0.93;1.98) | 0.11 |  |
| AA- | - |  | 2.32 (1.36;3.94) | 0.002 |  |
| AA+ | - |  | 0.84 (0.80;1.46) | 0.53 |  |
|  |  |  |  |  |  |
| 19-Dislike | 1.46 (0.60;3.58) | 0.40 | 1.93 (1.22;3.06) | 0.005 |  |

Note: results shown only for items with significant associations after Bonferroni correction for Low and/or High DSL, or for DSL-specific AA- and/or AA+ categories

^1^for subjects with pre-dementia: a high versus low depressive symptomatology was defined as a CES-D score≥16 at assessment point or at any earlier follow-up, including study entry; for others: this was defined as a CES-D score≥16 at the 2, 4 or 7-year follow-up

^2^Cox proportional hazard model with age as the time-scale, adjusted for study centre, education (>5 years), Apoe4 and the following time-dependent variables: diabetes (no/yes), ischemic disease (no/yes), dependency (3 levels)
