# Supplementary material for: Sex-specific depressive symptoms as markers of pre-Alzheimer dementia: findings from the Three-City cohort study
Source: Transl Psychiatry. 2019 Nov 11;9:291. doi: 10.1038/s41398-019-0620-5 (PMC6848073; doi:10.1038/s41398-019-0620-5)
Supplement: Supplementary file 2 — Figure S1 [file 41398_2019_620_MOESM2_ESM.pptx]

## Slide 1
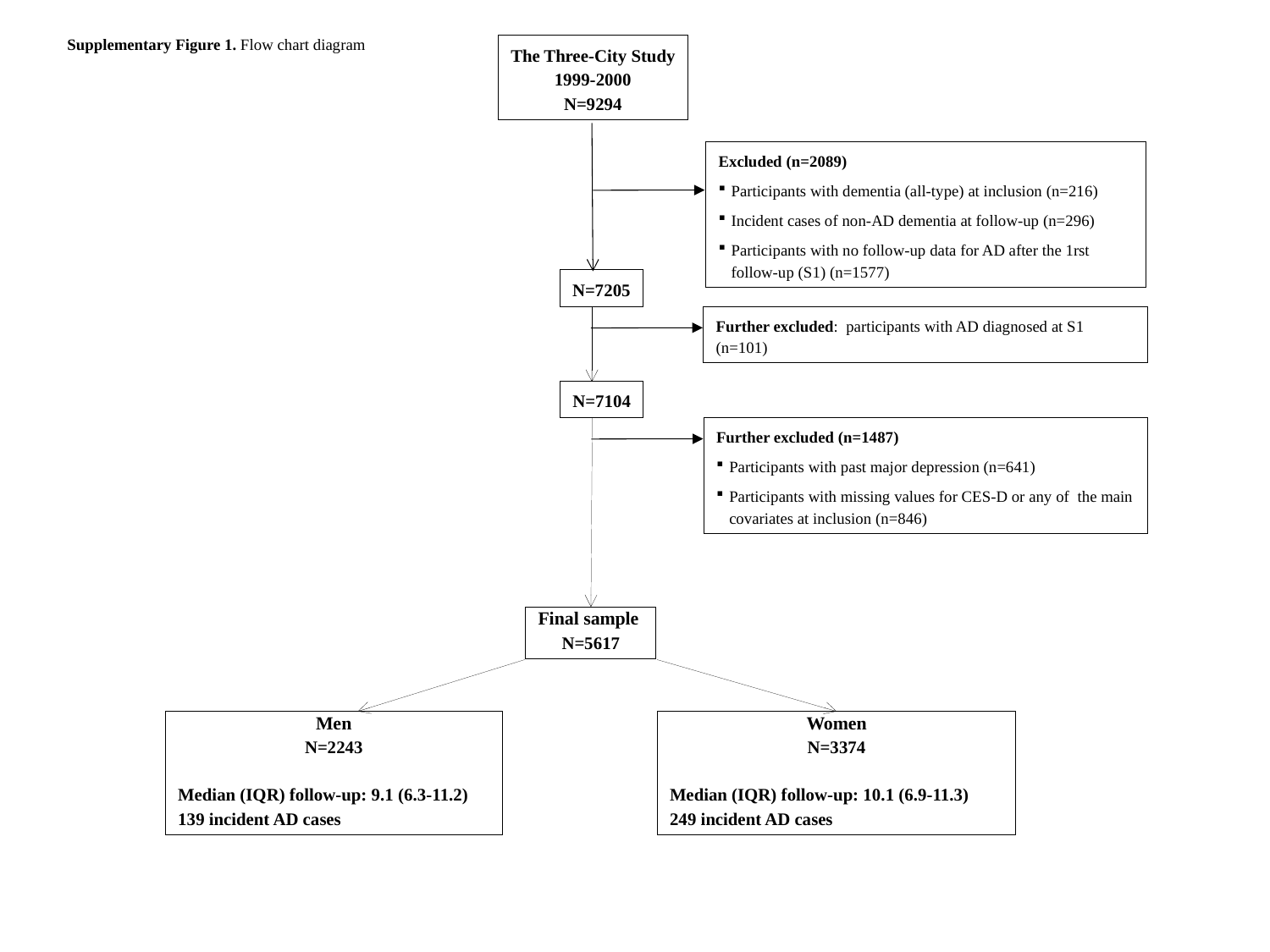

Supplementary Figure 1. Flow chart diagram
The Three-City Study
1999-2000
N=9294
Excluded (n=2089)
Participants with dementia (all-type) at inclusion (n=216)
Incident cases of non-AD dementia at follow-up (n=296)
Participants with no follow-up data for AD after the 1rst follow-up (S1) (n=1577)
N=7205
Further excluded: participants with AD diagnosed at S1 (n=101)
N=7104
Further excluded (n=1487)
Participants with past major depression (n=641)
Participants with missing values for CES-D or any of the main covariates at inclusion (n=846)
Final sample
N=5617
Women
N=3374
Median (IQR) follow-up: 10.1 (6.9-11.3)
249 incident AD cases
Men
N=2243
Median (IQR) follow-up: 9.1 (6.3-11.2)
139 incident AD cases
